# Patterns of use of antimuscarinic drugs to treat overactive bladder in Denmark, Sweden, and the United Kingdom

**DOI:** 10.1371/journal.pone.0204456

**Published:** 2018-09-27

**Authors:** Andrea V. Margulis, Marie Linder, Alejandro Arana, Anton Pottegård, Ina Anveden Berglind, Christine L. Bui, Nina Sahlertz Kristiansen, Shahram Bahmanyar, Lisa J. McQuay, Willem Jan Atsma, Kwame Appenteng, Milbhor D'Silva, Susana Perez-Gutthann, Jesper Hallas

**Affiliations:** 1 Pharmacoepidemiology and Risk Management, RTI Health Solutions, Barcelona, Spain; 2 Centre for Pharmacoepidemiology, Karolinska Institutet, Stockholm, Sweden; 3 Clinical Pharmacology and Pharmacy, Department of Public Health, University of Southern Denmark, Odense, Denmark; 4 Pharmacoepidemiology and Risk Management, RTI Health Solutions, Research Triangle Park, North Carolina, United States of America; 5 Astellas, Leiden, Netherlands; 6 Astellas, Northbrook, Illinois, United States of America; University of Oxford, UNITED KINGDOM

## Abstract

**Purpose:**

To describe the use of antimuscarinic drugs to treat overactive bladder (OAB) in Denmark, Sweden, and the United Kingdom (UK).

**Methods:**

We identified new users of darifenacin, fesoterodine, oxybutynin, solifenacin, tolterodine, and trospium aged 18 years or older from the Danish National Registers (2004–2012), the Swedish National Registers (2006–2012), and UK Clinical Practice Research Datalink (2004–2012). Users were followed until disenrollment, cancer diagnosis, death, or study end. Treatment episodes, identified by linking consecutive prescriptions, were described with respect to duration, drug switch, and drug add-on.

**Results:**

Mean age of OAB drug users was 66 years in Denmark (n = 72,917) and Sweden (n = 130,944), and 62 years in the UK (n = 119,912); 60% of Danish and Swedish patients and 70% of UK patients were female. In Denmark, of 224,680 treatment episodes, 39% were with solifenacin, and 35% with tolterodine; 2% were with oxybutynin. In Sweden, of 240,141 therapy episodes, 37% were with tolterodine and 35% with solifenacin; 5% were with oxybutynin. In the UK, of 245,800 treatment episodes, 28% were with oxybutynin, 27% with solifenacin, and 26% with tolterodine. In the three countries, 49%-52% of treatment episodes comprised one prescription and over 80% of episodes ended because of no refill; less than 20% ended because of a switch to another antimuscarinic. During the study years, we observed a change in OAB treatment preference from tolterodine to solifenacin.

**Conclusions:**

In these cohorts, persistence with antimuscarinic drugs was low. By 2012, the preferred drug was solifenacin; oxybutynin use was marginal in Nordic countries compared with the UK.

## Introduction

According to the International Continence Society, overactive bladder (OAB) can be defined as urinary urgency, usually with urinary frequency and nocturia, with or without urgency urinary incontinence [1]. Using this definition, the self-reported prevalence of OAB was 13% in women and 11% of men in Canada, Germany, Italy, Sweden and the United Kingdom (UK) in 2005 [2]. Studies using non-standardised definitions have reported urgency incontinence prevalences as low as 2% and as high as 36% worldwide [3]. While treatment with antimuscarinic drugs has been reported to improve quality of life, [4] these medications are often discontinued due to side effects or limited efficacy.

The purpose of this study was to describe utilisation patterns of antimuscarinic drugs to treat OAB in three European populations (Denmark, Sweden, and the UK’s Clinical Practice Research Datalink [CPRD]) at the time when only antimuscarinic drugs were available for the treatment of OAB; it was conducted to partially fulfil a regulatory requirement. This study updates previous reports on the use of these drugs in Denmark and the UK [5–7].

## Materials and methods

### Ethics statement

This study was judged to be exempt from review by the RTI International institutional review board and was approved by the Ethical Review Board at Karolinska Institutet, Stockholm (Ref no.2014/1478-31). In Denmark, pure register studies are exempt from review by an Ethics Committee. For this type of study patient consent is not required.

### Setting

We conducted a cohort study including adults newly exposed to antimuscarinic drugs used to treat OAB from January 1, 2004, through December 31, 2012, in Denmark and the UK. In Sweden, the study period started on January 1, 2006 (1 year after dispensed prescriptions started to be recorded in the Swedish Prescribed Drug Register), and also finished on December 31, 2012. The study drugs were darifenacin, fesoterodine, oxybutynin, solifenacin, tolterodine, and trospium (trospium was not available in Sweden; codes are provided in the S1 Table). The diagnosis of OAB was not required for inclusion in the study. In Denmark and Sweden, we ascertained exposure through dispensed prescriptions, and in the UK with issued prescriptions. In each country, the study population included all patients aged 18 years or older with at least 12 months of continuous enrolment in the database followed by a prescription for a study drug, provided that the same drug was not prescribed during the previous 12 months. The first recorded prescription that met this criterion was the patient’s index prescription. Patients with cancer (except non-melanoma skin cancer) diagnosed prior to the index prescription were excluded. In Sweden and the UK, subjects with HIV infection prior to the index prescription were also excluded. This was done in the UK because health care may occur outside the routine avenues after this diagnosis, and thus health care may not be fully captured. Sweden applied this criterion for consistency across data sources. Follow-up started with the index prescription and ended at the earliest of the end of the study period, cancer diagnosis (except non-melanoma skin cancer), death, emigration, or disenrollment from the database. During the study period, the included drugs were authorised for sale only by prescription in the three countries.

### Data sources

In the three countries, we used health information collected during routine health care. In Denmark, data were collected from the following nationwide registers: the Danish Civil Registry (dates of birth, death, and migration) [8], the Danish National Registry of Patients (hospital diagnoses) [9], the Danish Cancer Registry (cancer diagnoses) [10], the Cause of Death Registry [11], and the Danish National Prescription Registry (DNPR). The DNPR contains data on all drugs dispensed to Danish residents since 1995, prescribed by general practitioners or specialists [12]. The data include information on substance name, brand name, pharmaceutical form, dispensed quantity of the drug, and date of dispensing. Information on over-the-counter drugs, drugs administered at hospitals or nursing homes, or drugs prescribed but not dispensed is not recorded in the DNPR.

Swedish nationwide registers used for this study were the Swedish National Patient Register (hospital diagnoses) [13], the Swedish Cancer Register [14], the Swedish Cause of Death Register [15], and the Swedish Prescribed Drug Register [16]. The latter includes Anatomical Therapeutic Chemical (ATC) code, brand name, pharmaceutical form, strength, package size, number of packages dispensed, dispensed number of defined daily doses [17], and prescribing and dispensing dates. The register does not include over-the-counter medicines, medicines administered at hospitals and nursing homes, or medicines prescribed but not dispensed.

In the UK, we used data from CPRD GOLD (primary care electronic medical records), Hospital Episode Statistics (hospital diagnoses), National Cancer Data Repository, and Office for National Statistics (mortality data). Drug use was ascertained from prescriptions issued by general practitioners; information includes the substance name, brand name, pharmaceutical form, strength, British National Formulary coding, dose, and days’ supply.

### Statistical analysis

Treatment episodes were identified by linking consecutive prescriptions into a single treatment episode, with slightly different implementations in each country. In Denmark, we assumed that two prescriptions with dispensing dates falling within a drug-specific interval were part of the same treatment episode. The duration of these intervals was estimated through a waiting-time distribution analysis, which is a modelling based on the observed intervals between dispensings. We constructed these waiting-time–based models specifically for each drug [18, 19]. Switching and add-ons were defined by an overlap between treatment episodes for two different OAB drugs. Overlaps longer than 60 days were considered add-ons; shorter overlaps were considered switches.

In Sweden, therapy episodes were defined based on adjacent dispensings for the same drug with a gap ≤ 60 days between the end of a dispensing and the beginning of the following dispensing. The duration of dispensings was set to the number of tablets, or 7 × (number of patches)/2 or (dispensed volume of solution)/30 mL. Dispensings longer than 14 days were extended by 7 days to allow for nonadherence. Dispensings of a different OAB drug within 7 days of an ongoing dispensing or dispensings that ended during an ongoing dispensing were considered add-ons. All other dispensings of a second OAB drug during current OAB drug use were classified as switches.

In the UK, therapy episodes included adjacent prescriptions for the same drug as long as the gap between the end of a prescription and the beginning of the following prescription did not exceed 60 days. When the days-of-supply field for a prescription was missing or equal to zero or days’ supply could not be calculated from other variables on the prescription record, the days’ supply was imputed as the modal days’ supply for all prescriptions in the study cohort of the same study drug and strength with nonmissing and nonzero days’ supply. Each therapy episode was extended by 7 days to allow for nonadherence. Add-ons occurred when patients started taking another OAB drug while continuing the current OAB therapy. Switches occurred when patients stopped taking a OAB drug and started taking a different OAB drug in an adjacent therapy episode, or when patients on more than one OAB drug dropped one or more of those drugs while continuing to take the other drug(s).

In the three countries, episodes could end due to lack of refill, switch to another drug, addition of another antimuscarinic drug (“add-on”), or end of follow-up.

We described characteristics of patients and treatment episodes. Patient characteristics were assessed using all available data before cohort entry. Trends in drug use were shown as the proportion of patients who entered the cohort on each drug each year (e.g., of the 5,122 patients who entered the cohort in 2004 in Denmark, 3,679 were on tolterodine, representing 72% of the new users of antimuscarinics that year).

This study was registered in the EU PAS Register before the start of data collection (Danish component: EUPAS8441, Swedish component: EUPAS8444, UK component: EUPAS5529) [20].

## Results

### Denmark

**Fig 1:**
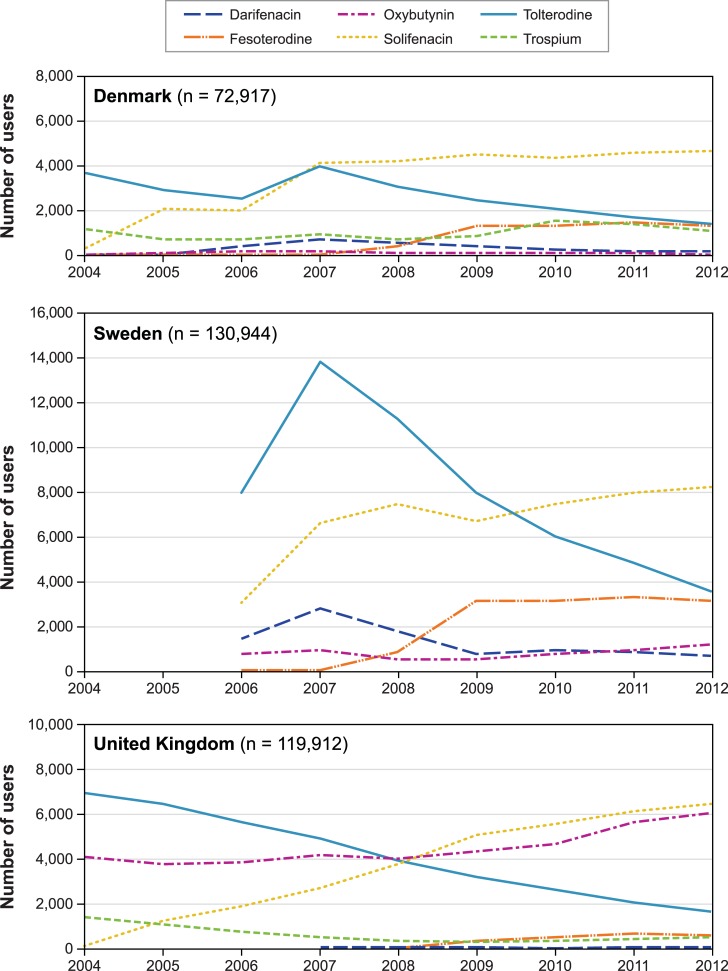
Trends in new use of antimuscarinic drugs for overactive bladder in Denmark, Sweden, and the United Kingdom^a a^ Displayed are the proportions of patients who entered the cohort with each drug each year.

All study drugs were available as tablets. The cohort included 72,917 patients with a mean age of 66 years, of whom 60% were women (Table 1). Patient characteristics and morbidity were similar across drugs, except that patients who entered the cohort on oxybutynin (1%) had a higher proportion of women (81%) than other drugs. A total of 224,680 therapy episodes were observed (3.1 episodes per person on average) (Table 2); solifenacin accounted for 39% of them and tolterodine for 35%. Over the study period, the number of new users of OAB antimuscarinics increased from 2004 to 2007 (5,122 to 9,969 new users). By 2012, the number had decreased to 8,725. Over these years, there was a decrease of new use of tolterodine and an increase of new use of solifenacin (Fig 1).

**Table 1:** Patient characteristics by index antimuscarinic OAB drug in Denmark, Sweden, and the UK.

Characteristic	All		Darifenacin		Fesoterodine		Oxybutynin		Solifenacin		Tolterodine		Trospium	
	n	%	n	%	n	%	n	%	n	%	n	%	n	%
**Denmark**														
Patients	72,917		2,698	3.7	5,749	7.9	740	1.0	30,792	42.2	23,776	32.6	9,105	12.5
Age in years (median, IQR)	66 (mean)	15.1 (SD)	69	59–78	67	56–76	66	55–76	68	57–77	69	58–78	68	56–77
Women	43,434	59.6	1,768	65.5	3,407	59.3	597	80.7	18,353	59.6	13,812	58.1	5,466	60.0
Hypertension^a^	16,051	22.0	561	20.8	1,416	24.6	175	23.6	6,974	22.6	5,012	21.1	1,903	20.9
Diabetes^a^	5,773	7.9	207	7.7	500	8.7	55	7.4	2,413	7.8	1,881	7.9	713	7.8
Stroke	10,456	14.3	353	13.1	719	12.5	121	16.4	4,179	13.6	3,806	16.0	1,268	13.9
Coronary heart disease	4,340	6.0	152	5.6	315	5.5	33	4.5	1890	6.1	1464	6.2	486	5.3
Heart failure	3,717	5.1	133	4.9	272	4.7	29	3.9	1508	4.9	1344	5.7	425	4.7
**Sweden**														
Patients	130,944		9,093	6.9	13,536	10.3	5,420	4.1	47,313	36.1	55,510	42.4	n/a	
Age in years (mean, SD)	66	15.3	67	14.2	65	14.5	55	20.2	65	14.8	68	15.1	n/a	
Women	77,992	59.6	5,748	63.2	8,075	59.7	3,409	62.9	30,457	64.4	30,259	54.5	n/a	
Hypertension^a^	62,492	47.7	4,331	47.6	6,448	47.6	1,905	35.1	22,385	47.3	27,391	49.3	n/a	
Diabetes^a^	14,732	11.3	1,010	11.1	1,532	11.3	455	8.4	5,185	11.0	6,545	11.8	n/a	
Stroke	7,445	5.7	563	6.2	724	5.3	159	2.9	2,322	4.9	3,673	6.6	n/a	
Coronary heart disease	12,699	9.7	995	10.9	1,326	9.8	367	6.8	4,270	9.0	5,734	10.3	n/a	
Heart failure	5,029	3.8	380	4.2	499	3.7	115	2.1	1,630	3.4	2,401	4.3	n/a	
**UK**														
Patients	119,912		151	0.1	2,344	2.0	40,651	33.9	33,120	27.6	37,506	31.3	6,071	5.1
Age in years (mean, SD)	62.4	16.7	65.3	14.4	60.1	16.5	62.8	17.4	61.3	16.3	62.8	16.3	64.1	16.1
Women	83,734	69.8	106	70.2	1,642	70.1	27,515	67.7	24,476	73.9	25,740	68.6	4,203	69.2
Smoking^b^														
Never	56,788	47.4	72	47.7	1,098	46.8	19,050	46.9	15,622	47.2	18,018	48.0	2,896	47.7
Former	42,229	35.2	52	34.4	871	37.2	14,448	35.5	12,043	36.4	12,702	33.9	2,093	34.5
Current	19,451	16.2	25	16.6	372	15.9	6,597	16.2	5,313	16.0	6,164	16.4	963	15.9
Hypertension^a^	96,738	80.7	126	83.4	1,872	79.9	32,873	80.9	26,645	80.4	30,170	80.4	4,990	82.2
Diabetes^a^	13,495	11.3	16	10.6	300	12.8	4,711	11.6	3,864	11.7	3,862	10.3	734	12.1
Stroke	8,309	6.9	15	9.9	172	7.3	2,984	7.3	2,044	6.2	2,594	6.9	497	8.2
Coronary heart disease	15,541	13.0	25	16.6	285	12.2	5,309	13.1	4,034	12.2	4,964	13.2	915	15.1
Heart failure	3,869	3.2	9	6.0	64	2.7	1,438	3.5	863	2.6	1,259	3.4	234	3.9

COPD, chronic obstructive pulmonary disease; IQR, interquartile range; n/a, not applicable; OAB, overactive bladder; SD, standard deviation; UK, United Kingdom. Note: 57, 72, and 69 patients entered the cohort on multiple drugs in Denmark, Sweden, and the UK, respectively (included in the column for all drugs combined). Counts < 5 subjects not shown. Patient characteristics were assessed using all available data before cohort entry.

^a^ Based on diagnosis or treatment.

^b^ 1.2% of 119,912 patients had unknown smoking status.

**Table 2:** Characteristics of therapy episodes by therapy episode drug in Denmark, Sweden, and the UK.

Characteristic	All		Darifenacin		Fesoterodine		Oxybutynin		Solifenacin		Tolterodine		Trospium	
	n	%	n	%	n	%	n	%	n	%	n	%	n	%
**Denmark**														
Therapy episodes^a^	224,680		9,383	4.2	20,172	9.0	3,616	1.6	87,312	38.9	77,970	34.7	26,227	11.7
Prescriptions per episode^b^														
1	110,607	49.2	4,013	42.8	9,883	49.0	1,827	50.5	39,861	45.7	41,826	53.6	13,197	50.3
2	43,544	19.4	1,951	20.8	4,092	20.3	655	18.1	17,671	20.2	15,259	19.6	3,916	14.9
3	21,095	9.4	1,041	11.1	1,832	9.1	330	9.1	9,328	10.7	6,239	8.0	2,325	8.9
4	11,858	5.3	626	6.7	1,100	5.5	199	5.5	5,458	6.3	3,076	3.9	1,399	5.3
≥ 5	37,576	16.7	1,752	18.7	3,265	16.2	605	16.7	14,994	17.2	11,570	14.8	5,390	20.6
Therapy episodes ending in a switch to or an add-on with														
Any drug^c^	15,669	7.0	1,026	10.9	1,556	7.7	855	23.6	4,926	5.6	4,608	5.9	2,698	10.3
Darifenacin^d^	1,178	7.5	n/a		114	7.3	103	12.0	432	8.8	314	6.8	215	8.0
Fesoterodine^d^	2,559	16.3	153	14.9	n/a		112	13.1	1,200	24.4	625	13.6	469	17.4
Oxybutynin^d^	1,123	7.2	98	9.6	157	10.1	n/a		458	9.3	244	5.3	166	6.2
Solifenacin^d^	4,827	30.8	357	34.8	658	42.3	313	36.6	n/a		2,440	53.0	1,059	39.3
Tolterodine^d^	3,264	20.8	239	23.3	291	18.7	200	23.4	1,745	35.4	n/a		789	29.2
Trospium^d^	2,718	17.3	179	17.4	336	21.6	127	14.9	1,091	22.1	985	21.4	n/a	
**Sweden**														
Index therapy episodes	130,944		9,093	6.9	13,536	10.3	5,420	4.1	47,313	36.1	55,510	42.4	n/a	
Prescriptions per index therapy episode														
1	68,170	52.1	4,338	47.7	6,800	50.2	3,371	62.2	23,143	48.9	30,460	54.9	n/a	
2	19,275	14.7	1,527	16.8	1,975	14.6	747	13.8	7,268	15.4	7,746	14.0	n/a	
3	9,773	7.5	799	8.8	1,077	8.0	380	7.0	3,705	7.8	3,810	6.9	n/a	
4	6,963	5.3	511	5.6	751	5.6	239	4.4	2,749	5.8	2,713	4.9	n/a	
≥ 5	26,763	20.4	1,918	21.1	2,933	21.7	683	12.6	10,448	22.1	10,781	19.4	n/a	
Previous exposure to study drugs^e^	16,438	12.6	2,071	22.8	1,974	14.6	1,121	20.7	5,971	12.6	5,276	9.5	n/a	
Therapy episodes	240,141		17,989	7.5	30,570	12.7	11,813	4.9	83,222	34.7	88,844	37.0	n/a	
Therapy episodes ending in a switch to or an add-on with														
Any drug	39,894	16.6	4,449	24.7	4,575	15.0	2,789	23.6	10,611	12.8	14,262	16.1	n/a	
Darifenacin	4,244	10.6	n/a		454	1.5	414	3.5	1,497	1.8	1,612	1.8	n/a	
Fesoterodine	9,943	24.9	1,143	6.4	n/a		567	4.8	3,979	4.8	3,843	4.3	n/a	
Oxybutynin	3,631	9.1	453	2.5	562	1.8	n/a		1,235	1.5	1,054	1.2	n/a	
Solifenacin	13,317	33.4	1,660	9.2	2,288	7.5	1,024	8.7	n/a		7,625	8.6	n/a	
Tolterodine	7,689	19.3	1,163	6.5	1,242	4.1	763	6.5	3,807	4.6	n/a		n/a	
**UK**														
Index therapy episodes	119,912		140	0.1	2,238	1.9	39,994	33.4	31,856	26.6	36,777	30.7	5,543	4.6
Prescriptions per index therapy episode														
1	61,145	51.0	57	40.7	1,028	45.9	22,570	56.4	14,547	45.7	18,340	49.9	2,880	52.0
2	15,682	13.1	13	9.3	347	15.5	5,122	12.8	4,100	12.9	4,426	12.0	655	11.8
3	7,819	6.5	15	10.7	157	7.0	2,378	5.9	2,215	7.0	2,377	6.5	390	7.0
4	4,955	4.1	5	3.6	107	4.8	1,515	3.8	1,466	4.6	1,521	4.1	228	4.1
≥ 5	30,311	25.3	50	35.7	599	26.8	8,409	21.0	9,528	29.9	10,113	27.5	1,390	25.1
Previous exposure to study drugs^e^	5,730	4.8	11	7.9	85	3.8	517	1.3	707	2.2	708	1.9	402	7.3
Therapy episodes	245,800		741	0.3	6,782	2.8	69,581	28.3	65,466	26.6	63,407	25.8	14,308	5.8
Therapy episodes ending in a switch to or an add-on with														
Any drug	46,149	18.8	190	25.6	765	11.3	6,348	9.1	5,471	8.4	6,916	10.9	1,643	11.5
Darifenacin	483	1.0	n/a		13	1.7	52	0.8	91	1.7	57	0.8	18	1.1
Fesoterodine	3,364	7.3	21	11.1	n/a		290	4.6	922	16.9	351	5.1	112	6.8
Oxybutynin	9,964	21.6	45	23.7	188	24.6	n/a		1,911	34.9	2,153	31.1	434	26.4
Solifenacin	15,998	34.7	69	36.3	313	40.9	3,026	47.7	n/a		3,375	48.8	646	39.3
Tolterodine	10,258	22.2	24	12.6	107	14.0	2,396	37.7	1,634	29.9	n/a		431	26.2
Trospium	5,478	11.9	30	15.8	140	18.3	577	9.1	909	16.6	973	14.1	n/a	

n/a, not applicable; UK, United Kingdom. Note: For Denmark, therapy episodes with multiple drugs are included in the columns for each of the involved drugs. For Sweden and the UK, they are counted separately. For Denmark, we present information on number of prescriptions per therapy episode and drugs switched to or added based on all therapy episodes. For Sweden and the UK, we present information on number of prescriptions based on index therapy episodes and drugs switched to or added based on all therapy episodes.

^a^ The denominator for percentages in this row is the number of therapy episodes for all drugs combined, presented in the leftmost column (i.e., these are row percentages).

^b^ The denominator for percentages in the rows for number of prescriptions per therapy episode is the number of episodes for all drugs combined or each individual drug, as appropriate, presented in the same pair of columns (i.e., these are column percentages).

^c^ The denominator for percentages in this row is the number of therapy episodes for all drugs combined or each individual drug, as appropriate, presented in the same pair of columns (i.e., these are column percentages).

^d^ The denominator for percentages in these rows is the number of therapy episodes in which there was a switch or add-on for all drugs combined or each individual drug, as appropriate, presented in the same pair of columns (i.e., these are column percentages).

^e^ In the 12 months before the episode. This information is not available for Denmark.

Nearly half (49%) of the episodes comprised a single prescription; tolterodine had the largest proportion of single-prescription therapy episodes (54%) and the lowest proportion of episodes with five or more prescriptions (15%). Nearly all (93%) episodes ended due to lack of renew or refill. The most common drug switched to or added on was solifenacin. For solifenacin drug episodes, the most common drug switched to or added on was tolterodine.

### Sweden

Trospium was not available during the study period. All study drugs were available as tablets; oxybutynin was also available in transdermal patches and as intravesical solution. The cohort comprised 130,944 patients, with a mean age of 66 years, 60% women (Table 1). Patient characteristics and morbidity were generally similar across drugs, except that patients who entered the cohort on oxybutynin (4%) were younger on average (mean age 55 years, with a wide dispersion), and were somewhat healthier. We observed 240,141 therapy episodes (1.8 episodes per person on average) (Table 2): 37% with tolterodine and 35% with solifenacin. New use of the study drugs first increased from 13,223 users in 2006 to 24,173 in 2007, and then decreased to 16,618 new users in 2012. Over the study period, there was a net decrease in new use of tolterodine and an increase in new use of solifenacin and fesoterodine (Fig 1).

Of index therapy episodes, 52% had only one prescription (Table 2). Oxybutynin was the drug with the largest proportion of single-prescription therapy episodes (62%), the lowest proportion of episodes with five or more prescriptions (13%), and one of the highest prevalences of use of study drugs (excluding the study drug on which patients entered the cohort) in the 12 months before cohort entry (21%). Of all episodes, 83% ended because the prescription was not renewed or refilled. The most common drug switched to or added was solifenacin. For solifenacin treatment episodes, the most common drug switched to or added was tolterodine.

### United Kingdom

All study drugs were available as tablets; oxybutynin was also available in transdermal patches and oral solutions. The cohort comprised 119,912 new users of antimuscarinic OAB drugs with mean age 62 years, 70% women; 16% were current smokers and 35% were former smokers (Table 1). The distribution of patient characteristics and morbidity was similar across users of individual drugs.

Index therapy episodes consisted of a single prescription in 51% of episodes, with some variation across individual drugs (Table 2). Oxybutynin had the largest proportion of single-prescription therapy episodes (56%) and the lowest proportion of therapy episodes with five or more prescriptions (21%). About 5% of index therapy episodes occurred in patients who had used an antimuscarinic OAB medication (excluding the drug on which the patient entered the cohort) in the 12 months before cohort entry. Over the study period, we observed 245,800 therapy episodes (2 episodes per person on average). The three most commonly used drugs, oxybutynin, solifenacin and tolterodine, accounted for 81% of drugs in similar proportions (Table 2). New use of OAB drugs increased from 12,598 in 2004 to a maximum of 15,441 in 2012. New use of tolterodine decreased over the study period, while new use of oxybutynin and solifenacin increased (Fig 1).

Overall, 81% of all therapy episodes ended due to lack of renew or refill. For episodes with any individual drug but solifenacin, the most common drug switched to or added was solifenacin (Table 2). For solifenacin therapy episodes, the most common drug switched to or added was oxybutynin.

## Discussion

The study cohorts in Denmark, Sweden and the UK had similar distributions of age and sex, with a mean age at cohort entry of 66 years in Denmark and Sweden and 62 years in the UK. About 60% of the Danish and Swedish patients and 70% of UK patients were women. Patient characteristics were generally similar across drugs within each country. In all countries, there was a decreasing trend of new use of tolterodine and increasing new use of solifenacin. In Sweden, new use of fesoterodine also increased, and trospium was not available. While use of oxybutynin was low in Denmark and Sweden, it accounted for a third of therapy episodes in the UK, with an upward trend in new use. In the three countries, about half of index episodes had only one prescription, and over 80% of episodes ended due to lack of renew or refill. Solifenacin was the drug most commonly switched to or added.

The use of antimuscarinic OAB drugs has changed over time, as new drugs have become available and older drugs fall out of preference. In Denmark, based on publicly available data from the primary sector for users 20 years old or older, only tolterodine was available in year 2000, trospium became available in 2001 and use of darifenacin, oxybutynin, and solifenacin began later; all following a generally upward trend in use (http://www.medstat.dk/en). In our data, we observed a decrease in the use of tolterodine and an increase in the use of solifenacin. Trospium, widely used before, was a less preferred drug in our study. In Sweden, only oxybutynin and tolterodine were available in year 2000 [21]. Until 2006, the use of oxybutynin was relatively low, and the use of tolterodine and the newer solifenacin and darifenacin increased [21]. From 2006 onward, we observed in Sweden a continuation of those trends, with a peak number of new users of tolterodine in 2007 (a decreasing trend in the proportion of new users was already present) and an increase in use of solifenacin and fesoterodine. Use of oxybutynin was low in our study. In the UK, only oxybutynin was licensed for OAB treatment in 1987. Until 2002, oxybutynin was the most common OAB drug prescribed to patients with symptoms of OAB, and tolterodine was the most common second-line drug. From 2002, tolterodine was the most common first-line antimuscarinic OAB drug [22]. From 2004 onwards, we observed a decrease in the use of tolterodine, an increase in the use of solifenacin, and a slight increase in the use of oxybutynin.

This study was designed to capture patients’ first exposure to any study medication in patients who were 18 years old or older at any time of the study period. In 13% of index therapy episodes in Sweden and 5% in the UK, exposure to study drugs had been recorded in the 12 months before cohort entry (excluding the study drug on which they entered the cohort). The proportion of previous use of study drugs varied by drug and by country, probably reflecting changes in drug availability and prescribing preference in each country over time. Clinical guidelines did not consider any of the study drugs an ideal first-line treatment for all patients; guidelines recommended tailoring treatments to each patient’s clinical characteristics and concomitant medications [23]. Drugs included in this study were given the same level of evidence and recommendation in the 2010 Guidelines on Urinary Incontinence of the European Association of Urology [23]. During the study years, though, we observed a change in overactive bladder treatment preference from tolterodine to solifenacin, which was the preferred study drug by 2012, and oxybutynin use was marginal in the Nordic countries compared with the UK. We did not find consistent patterns of morbidity that would provide evidence of channeling of patients with a specific morbidity profile to a specific antimuscarinic drug.

Persistence with antimuscarinic drugs was low. In each of the three populations, slightly over 50% of the index therapy episodes consisted of a single prescription, in line with findings in a US claims study of patients enrolled in a regional managed care plan with prescription coverage from 1999 to 2003 [24]. In this study, 45% of patients did not refill their first prescription. In another US claims study that identified patients with OAB diagnoses who started antimuscarinic OAB treatment in 2005–2008, 33% of episodes consisted of a single prescription [25]. A third US claims study in patients with OAB diagnoses who started antimuscarinic OAB treatment in 2005–2008 reported that 30% of patients with diabetes and 36% of patients without diabetes stopped treatment after one prescription [26]. In Norway in 2004–2010, 32% of women who used OAB antimuscarinics filled only one prescription [27]. Studies evaluating persistence also noted discontinuation of our study drugs at 6 months of more than 50% in Denmark in 1999–2006 [5], 59% in the UK in 1991–2007 [6], and 54%-71% in the UK in 2007–2008 [7]. A study in Sweden that looked at users of OAB antimuscarinics in 2007–2008 (2 years in the middle of our study period) reported results similar to ours [28].

A strength of this study is the use of population-based databases covering a period of 9 years (7 in Sweden) and following a common protocol that was locally adapted to the nuances of each data source. Our data sources include practically the entire populations of Denmark and Sweden; CPRD reflects the structure of the UK population in terms of sex, age, and ethnicity [29]. The long study period enabled us to see trends in drug use; it remains to be seen how these change with the approval of new treatments with new mechanisms of action: mirabegron, an oral beta-3 adrenergic receptor agonist, and onabotulinumtoxinA, an injectable acetylcholine release inhibitor and neuromuscular blocking agent.

The main limitation of this study was that we did not have information on treatment discontinuation before the end of the prescribed amount, or on the reasons for discontinuation (side effects are common, and symptom control may require multiple trials of treatment options [4, 30]). The estimation of duration of prescriptions followed different processes in the three data sources because the common protocol outlined the estimation process, but allowed for flexibility so that researchers could apply the methods most appropriate for their data source. We found similar patterns of drug use in the three data sources, hinting that results might not be sensitive to the variation in methods. A limitation specific to the UK is that CPRD only includes prescriptions issued by general practitioners. However, most prescriptions for OAB should be captured, as UK´s National Institute for Health and Care Excellence (NICE) recommends referral to a specialist only after failure of the first or second OAB drug [31, 32].

In conclusion, in these three cohorts of new users of antimuscarinic drugs to treat OAB from Denmark, Sweden and the UK in years 2004–2012, we observed preference for OAB antimuscarinic treatment changing from tolterodine to solifenacin. Oxybutynin use was minimal in Denmark and Sweden compared with the UK. In all three countries, about half of the episodes consisted of one prescription. This finding was consistent with those from several other studies, reflecting that the pattern of use is common to the drug class, with some variation across individual drugs.

## Supporting information

S1 Table Supporting information. The file named S1_Table contains Table S1. Codes for Drugs to Treat Overactive Bladder.
(DOCX)
