# Supplementary material for: Patterns of use of antimuscarinic drugs to treat overactive bladder in Denmark, Sweden, and the United Kingdom
Source: PLoS One. 2018 Sep 27;13(9):e0204456. doi: 10.1371/journal.pone.0204456 (PMC6160033; doi:10.1371/journal.pone.0204456)
Supplement: S1 Table — The file named S1_Table contains Table S1. Codes for Drugs to Treat Overactive Bladder. (DOCX) [file pone.0204456.s001.docx]

# Supporting information

Patterns of use of antimuscarinic drugs to treat overactive bladder in Denmark, Sweden, and the United Kingdom

Margulis AV, Linder M, Arana A, Pottegard A, Anveden-Berglind I, Bui CB, Kristiansen NS, Bahmanyar S, McQuay LJ, Atsma WJ, Appenteng K, D´Silva M, Perez-Gutthann S, Hallas J.

Table S1. Codes for Drugs to Treat Overactive Bladder

| Denmark  (ATC codes) | Sweden  (ATC codes) ^a^ | United Kingdom  (Gemscript codes) | | |
| --- | --- | --- | --- | --- |
| G04BD04  G04BD07  G04BD08  G04BD09  G04BD10  G04BD11 | G04BD04  G04BD07  G04BD08  G04BD10  G04BD11 | 1956001  1956002  1703002  1703001  3180001  3093007  11099001  10831001  11099002  3180003  1703003  3180002  8161001  1521001  1956003  13136001  1521002  13137001  8159001  10676001  7545001  4632007  9910001  9910002  !5249901  11469001  11469002  11494001  2146010 | 2855010  2855009  2347009  2175009  2858009  2175010  1930009  3635009  2770010  2770009  2146009  2162009  5830009  20568020  20566020  33439020  11809001  11811002  11809002  11811001  11811003  11809003  !8503379  !8503380  16403001  35443020  7200020  35524020  35525020 | 7202020  44418020  45190020  35523020  7197020  14675001  14676001  14677001  14678001  12677001  12679001  12678001  12680001  37721020  8226001  9086001  17295001  17297001  17345001  39354020  7216020  41400020  41401020  16122001  16124001  16123001  16125001  11109020 |

**^a^** Trospium (ATC code G04BD09) was not available in Sweden.
